# Temporal Trends in Results Availability from Genome-Wide Association Studies

**DOI:** 10.1371/journal.pgen.1002269

**Published:** 2011-09-08

**Authors:** Andrew D. Johnson, Richard Leslie, Christopher J. O'Donnell

**Affiliations:** Division of Intramural Research and The Framingham Heart Study, National Heart, Lung, and Blood Institute, Framingham, Massachusetts, United States of America; Georgia Institute of Technology, United States of America

The application of genome-wide association study (GWAS) approaches for the study of genetic determinants of common diseases has propelled human genetics forward, resulting in a surfeit of genomic data. With the accompanying level of widespread collaboration and sharing of data, access to this body of valuable genomic data and the application of novel analytic approaches beyond the level of first GWAS scans is yielding additional insights, both in terms of new genetic discoveries and important general biological findings [1]. However, recent work shows that standard statistical approaches can be applied to aggregate genome-wide association results that place individual research participants at increased risks for misuse related to privacy and confidentiality. We define “misuse” as analysis efforts aimed at exposing individual research participants' information, including revealing disease status, predicted future likelihood or past presence of other traits, or attempts to link another DNA result with a participant, for example, to determine presence or absence in a research cohort, ancestry, and relatedness (e.g., paternity/non-paternity). Thus, there is the small but theoretically possible risk of later legal or discriminatory actions that were originally unforeseen by investigators and would likely be unwanted and unexpected by the research participants [2]–[7].

At this time the risks to research participant identification generally exist when there is access to (at least) a moderate number of genetic variant results that include both statistics (regression coefficients or two-sided *p*-values) and cohort-specific population allele frequencies [2]–[5], [7]. To date, scientific discussion about these potential risks has focused largely on theoretical scenarios and the related ethical and policy responses [2]–[8]. Initial publications [2], [3] resulted in significant policy shifts and reduction in the open access to GWAS results by the creation of controlled access repositories for results (e.g., for the Wellcome Trust Case Control Consortium [WTCCC] results and Framingham Heart Study [FHS] SHARe 100K GWAS results), but the literature contains no systematic assessment of the extent of current GWAS results availability, temporal trends in availability, or the number of studies that remain at a potentially unacceptable level of risk.

## Design of Survey of Results Availability from 643 GWASs

We conducted a systematic and chronologic survey of 643 GWAS articles published between November 2002 and July 1, 2010. Studies were identified from our past GWAS database effort [1] and through the National Human Genome Research Institute (NHGRI) catalog (http://www.genome.gov/26525384), updated and supplemented by a controlled vocabulary search of PubMed using QUOSA (v. 8.06.631, Waltham, MA). Articles were retrieved by PubMed ID using QUOSA. Abstracts and articles were scanned to identify GWAS analyses as opposed to other categories such as linkage studies or studies attempting to replicate GWAS. Linkage studies and large-scale candidate gene studies were not included here, though these may also carry similar risks if they expose large numbers of marker results. Studies that reported only conducting copy number variation (CNV)-based analysis were excluded (*n* = 5). Publication dates were determined via the NHGRI GWAS catalog, PubMed, or the individual publications, selecting the earliest known date of availability. Complete supplementary materials for all 643 GWAS articles were downloaded from journal websites or from independent websites cited in the publications.

We also sought access to supplemental data sources if such access required a limited application without the additional requirement of an Institutional Review Board approval or other extensive approvals. Information was recorded on the mode of access and the type of data that was accessible through either open access (via the Internet or journal content) or through a controlled access process. If results were available only by an application process, we contacted the corresponding author to obtain updated information. We noted all instances for which the mode of data access changed compared with the access process that was stated in the original publication.

For each GWAS manuscript, we estimated the amount and type of results available either by open access or by a controlled access mechanism. An individual SNP result, hereafter termed “SNP-specific result”, refers to an association test result with a single SNP unique within that publication or its supplements. We placed studies in one of five categories of increasing “identifiability risk”, based on recent recommendations regarding safe levels of data sharing [7]: category *0,* no SNP-specific results; category *1,* 1–10 SNP-specific results; category *2,* 11–499 SNP-specific results; category *3,* 500 or more SNP-specific results but not full dataset level results available; or category *4,* all SNP-specific results available or potentially available. If by reviewing the manuscript and supplemental materials we could not reliably estimate the appropriate category, we extracted all available results to determine the exact number of results available. For each study we noted the type of results available at corresponding amounts of SNP-specific results (e.g., study-specific allele frequencies, individual genotypes, regression estimates, *p*-values).

**Figure 1:**
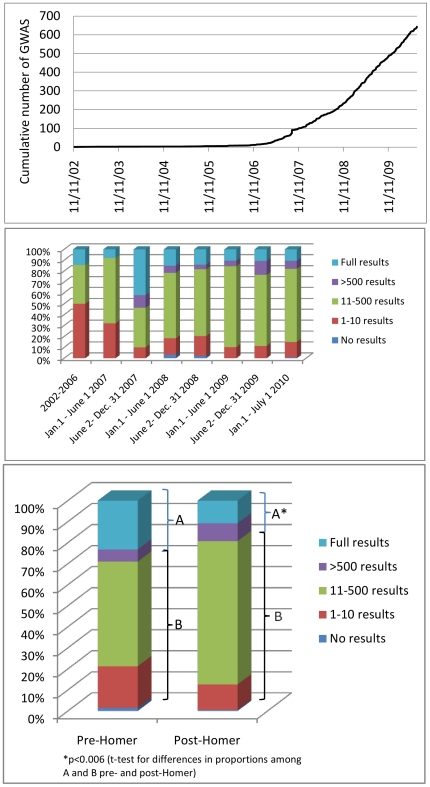
Temporal trends in GWAS publications and results availability. Top panel: Cumulative chronological release of 643 GWAS papers at their earliest release dates. Middle panel: Distribution of GWAS papers among five categories of reported maximum results availability from 2002 to 2006, and in 6-month intervals from January 1, 2007 to July 1, 2010. Bottom panel: Distribution of 643 GWAS papers among five categories of reported maximum results availability by open access or controlled access application in the period before October 1, 2008 (left) and from October 1, 2008 to July 1, 2010 (right).

Few GWA studies were published between 2002 and 2006, but the number of published GWA studies began to grow rapidly in 2007 (Figure 1). We identified 643 studies in the time period examined (November 1, 2002–July 1, 2010). For the same time period, a commonly referenced resource, the NHGRI GWA catalog (as of August 24, 2010), compiles 614 GWAS studies. Ten studies from the NHGRI GWAS catalog were not included in our study because they focused only on CNV analysis (*n* = 5), or because they represented re-analyses of previously published GWAS results (*n* = 5). Thus, we identified 39 additional studies in the same time period, compared with the NHGRI catalog, suggesting we have identified nearly all of the published GWA studies in the time period examined.

## Data Release Mechanisms and Temporal Trends in GWAS Results Sharing

The number of SNP-specific results that were made directly available upon publication of the original paper varied over a wide range, from zero (*n* = 5) to millions of results. Taken as the maximum number of SNP-association results made available, including SNP results made available by a controlled access application process such as dbGAP, we observed the following distribution of papers across the categories defined in the Methods section: category 0 (*n* = 5; 0.8%), category 1 (*n* = 94; 14.6%), category 2 (*n* = 400; 62.2%), category 3 (*n* = 49; 7.6%), category 4 (*n* = 95; 14.8%).

A number of studies reported the availability of SNP-specific genotypes for individual research participants, usually through a controlled access application process; however, seven studies were identified for which individual level genotype data was freely available at some point after publication. Of these seven studies, three required a minimal application to access genotypes, whereas four did not. One study made genotypes available for download for only ∼100 markers. Another study presented genotypes via a web browser interface that made it time-consuming to download genotypes for large numbers of markers. For two of the studies that initially made individual genotypes available through a minimal application process, the results no longer appear to be publicly available.

We selected a cutoff date of October 1, 2008 to define a period (“post-Homer”) after publication of the Homer et al. [2] report. We defined this period by allowing approximately 1 month before this paper likely impacted data sharing decisions in publications. Comparing the stated results availability in the original publications in time periods pre- and post-October 1, 2008, we find that a smaller proportion of studies provided access to extensive (category 3) or full (category 4) results in the later time period (Figure 1, *p<*0.006). While there is a slight contraction in the proportion of studies indicating possible access to large numbers of SNP-association results, there is a corresponding slight increase in the proportion of studies offering access to moderate or greater numbers of results (category 2, 3, or 4). This is observed in the relative growth over time (observed in 6-month intervals) of the proportion of studies in category 2 or 3 or 4 as opposed to the proportion in categories 0 or 1 (Figure 1).

For those studies originally indicating access to extensive (≥500 SNP results, category 3, *n* = 49) or full results (category 4, *n* = 95) at any time period (total *n* = 144 studies), we further examined the mode by which results were *currently* available as of July 15, 2010 to gauge mechanisms of access and whether there was indication that any studies had changed the availability of data after their initial publication. Eighty-four of 144 studies (58.3%) provided results that were either freely accessible by open Internet access or by a journal subscription. The remainder of the studies now require formal applications for data through some form of controlled access and/or have results that are no longer available via the original cited mechanism (*n* = 11). We assessed the number of studies for which there was evidence that data access models had changed since the original publication, and we found 35/144 studies (24.3%) appear to have modified the level of data access, in each case making access more restrictive.

Finally, we assessed the number of studies at potential risk for misuse given a current recommended guideline of fewer than 500 SNP-specific statistics without provision of allele frequency information [7]. Under these guidelines, we find that 44/643 studies (6.8%) may be at some level of risk as of July 15, 2010. Under a more conservative interpretation that assumes that allele frequency or regression statistics may be unnecessary for some risk for misuse, we find that up to 79/643 studies (12.3%) may be at potential risk.

## Research and Policy Implications of GWAS Results Availability

While extensive availability of GWAS results may provide a small but real threat to the privacy and confidentiality of research participants, tight restrictions on access to research results may inhibit dissemination for the legitimate, scientific use of these results [2], [6], [8]. Aggregate GWAS results that are made available are often re-used and cited as scientific support data [1]. This practice is common in many areas, and is well exemplified by re-uses of data from the WTCCC, Diabetes Genetic Initiative (DGI), and the FHS. The uses of available GWAS results are wide-ranging and include the further replication of known or novel genetic signals, the construction of reference control samples when such data are not otherwise easily available, development of novel methodological approaches to data analysis, and, increasingly, the search for evidence of pleiotropic associations for specific loci, to gain insight into the potential physiological mechanisms underlying the associations.

Since an initial effort raised privacy concerns [2], additional efforts have refined methodological approaches [3]–[5] and set boundaries on the methods [4], [7] that may be used to identify individual participants and participant disease status in GWAS results. Discussions have focused on nuanced issues that could arise and potential policy implementations to address them [6], [8]. In our survey of chronological GWAS results availability from a consecutive series of 643 published studies, a large majority of studies (87.7%–93.2%) appear to be in line with current recommendations [7]. These figures may over-estimate the proportion of studies at risk since some studies classified as “at potential risk” may have features that make misuse more difficult, e.g., large sample sizes with meta-analysis, combined population allele frequency statistics only, linkage disequilibrium between markers, and lack of inclusion of allele frequencies or regression statistics [4], [5]. The provision of summary statistics with minimized or homogenized information reduces risks for misuse. It is also important to note that we used one of the most recent suggested definitions of risk as greater than 500 SNP-specific results [7]. However, a single true threshold is unknown and depends on the circumstances of datasets included, methods applied, and intended use, and thresholds for risk may further evolve as new methods are developed. A significant number of studies that were at potential risk given their original reported mode of data access implemented more restrictive measures subsequent to their initial publication. Thus, concerns raised “post-Homer” have been accompanied by measurable decisions that were voluntarily made by scientists and/or journals.

Nonetheless, we did identify a minority of studies that seem to be at significant risk for potential misuse. Most alarming are a handful of studies where individual participant-specific genotypes are available publicly. We also identified several instances where research groups gained access to primary GWAS results and secondarily reported large numbers of SNP-association results, potentially exposing the study participants at unsafe levels. While data use certifications (DUCs) or access applications (DAAs) generally specify that users with data access should protect individual confidentiality, not attempt to identify individuals, and not sell or share controlled-access data, they are less specific about how data products (e.g., derived SNP-specific statistics) can be appropriately shared or published. It is difficult to ascertain whether the secondary posting we found violated any data use agreements or was in fact conducted without knowledge of the data use agreements; however, these examples illustrate that, even with data access protections in place, there will always be a potential risk of re-posting of results by third parties. This suggests that clearer guidance regarding appropriate disclosure of derived data is needed in DUCs and DAAs. Once results are posted publicly, they cannot be deemed safe even if posted results are eventually retracted, since backups may have been created. In our opinion, controlled access models have decreased the overall risk for results misuse across studies, but the examples of re-posting uncovered illustrate that controlled access is not fool-proof.

Given the small, but significant, number of studies currently at risk for misuse, our study provides evidence that the concerns raised about GWAS results misuse are indeed relevant to current reporting procedures for GWAS results. The increasing use of massively parallel next generation sequencing technology to conduct whole-exome, whole-genome, and whole-transcriptome sequencing studies for common diseases in large populations will provide a larger set of low frequency and private genetic variants that may allow easier identification of individual participants in research studies [9]. Direct chromosomal phasing of sequenced haplotypes could also increase the potential for individual identification. Deep sequencing promises valuable new research results, but the posting of aggregate sequencing-derived results may create risks for misuse. Risks for identification may increase with deep sequencing of families with highly penetrant disease or in populations that are discrete geographically or ethnically. Thus, research regarding risks of identifiability and guidelines for data sharing should also be considered urgently for the rapidly accumulating body of genome-wide sequencing data in large populations.

## References

[1] Johnson AD, O'Donnell CJ (2009). An open access database of genome-wide association results.. BMC Med Gen.

[2] Homer N, Szelinger S, Redman M, Duggan D, Tembe W (2008). Resolving individuals contributing trace amounts of DNA to highly complex mixtures using high-density SNP genotyping microarrays.. PLoS Genet.

[3] Jacobs KB, Yeager M, Wacholder S, Craig D, Kraft P (2009). A new statistic and its power to infer membership in a genome-wide association study using genotype frequencies.. Nat Gen.

[4] Visscher PM, Hill WG (2009). The limits of individual identification from sample allele frequencies: theory and statistical analysis.. PLoS Genet.

[5] Braun R, Rowe W, Schaefer C, Zhang J, Buetow K (2009). Needles in the haystack: identifying individuals present in pooled genomic data.. PLoS Genet.

[6] Church G, Heeney C, Hawkins N, de Vries J, P^3^G Consortium (2009). Public access to genome-wide data: five views on balancing research with privacy and protection.. PLoS Genet.

[7] Lumley T, Rice K (2010). Potential for revealing individual-level information in genome-wide association studies.. JAMA.

[8] Heeney C, Hawkins N, de Vries J, Boddington P, Kaye J (2010). Assessing the privacy risks of data sharing in genomics.. Pub Health Gen.

[9] Altshuler D, Durbin RM, Abecasis GR, Bentley DR, The 1000 Genomes Project Consortium (2010). A map of human genome variation from population-scale sequencing.. Nature.

